# Behavioral Adaptations of Nursing Brangus Cows to Virtual Fencing: Insights from a Training Deployment Phase

**DOI:** 10.3390/ani13223558

**Published:** 2023-11-17

**Authors:** Shelemia Nyamuryekung’e, Andrew Cox, Andres Perea, Richard Estell, Andres F. Cibils, John P. Holland, Tony Waterhouse, Glenn Duff, Micah Funk, Matthew M. McIntosh, Sheri Spiegal, Brandon Bestelmeyer, Santiago Utsumi

**Affiliations:** 1 Division of Food Production and Society, Norwegian Institute of Bioeconomy Research (NIBIO), PB 115, N-1431 Ås, Norway; 2 Department of Animal and Range Sciences, New Mexico State University, Las Cruces, NM 88003, USA; arcox@nmsu.edu (A.C.); arperea@nmsu.edu (A.P.); glennd@nmsu.edu (G.D.); funkm@nmsu.edu (M.F.); 3 United States Department of Agriculture-Agriculture Research Service, Jornada Experimental Range, Las Cruces, NM 88003, USA; rick.estell@usda.gov (R.E.); mattmac@nmsu.edu (M.M.M.); sheri.spiegal@usda.gov (S.S.); brandon.bestelmeyer@usda.gov (B.B.); 4 United States Department of Agriculture Southern Plains Climate Hub, United States Department of Agriculture-Agriculture Research Service, Oklahoma and Central Plains Agricultural Research Center, El Reno, OK 73036, USA; andres.cibils@usda.gov; 5 SRUC Hill and Mountain Research Centre, Scotland’s Rural College, Kirkton Farm, Crianlarich, Perthshire FK20 8RU, UK; john.holland@sruc.ac.uk (J.P.H.); tony.waterhouse@sruc.ac.uk (T.W.)

**Keywords:** virtual fence, conditional behavior, animal tracking, precision livestock farming, GPS location, accelerometer and activity

## Abstract

**Simple Summary:**

The study explores the use of virtual fencing technology for managing livestock distribution, focusing on nursing Brangus cows. The study investigates how these animals learn to avoid restricted areas and increase their reliance on auditory cues over time. The findings support the effectiveness of virtual fencing in controlling cow spatial behavior and highlight their ability to adapt to virtual boundaries rapidly. The research also presents a safe and efficient training protocol for implementing virtual fence systems.

**Abstract:**

Virtual fencing systems have emerged as a promising technology for managing the distribution of livestock in extensive grazing environments. This study provides comprehensive documentation of the learning process involving two conditional behavioral mechanisms and the documentation of efficient, effective, and safe animal training for virtual fence applications on nursing Brangus cows. Two hypotheses were examined: (1) animals would learn to avoid restricted zones by increasing their use of containment zones within a virtual fence polygon, and (2) animals would progressively receive fewer audio-electric cues over time and increasingly rely on auditory cues for behavioral modification. Data from GPS coordinates, behavioral metrics derived from the collar data, and cueing events were analyzed to evaluate these hypotheses. The results supported hypothesis 1, revealing that virtual fence activation significantly increased the time spent in containment zones and reduced time in restricted zones compared to when the virtual fence was deactivated. Concurrently, behavioral metrics mirrored these findings, with cows adjusting their daily travel distances, exploration area, and cumulative activity counts in response to the allocation of areas with different virtual fence configurations. Hypothesis 2 was also supported by the results, with a decrease in cueing events over time and increased reliance with animals on audio cueing to avert receiving the mild electric pulse. These outcomes underscore the rapid learning capabilities of groups of nursing cows in responding to virtual fence boundaries.

## 1. Introduction

Virtual fencing has emerged as a promising commercialized solution for efficiently managing livestock distribution, introducing an innovative paradigm for controlling their movements [1,2]. This method employs collars equipped with auditory-electric cues, designed to deter animals from crossing predetermined virtual fence boundaries [3,4]. These smart collars, integrated with online embedded micro-controllers, calculate the animal’s GPS position, travel direction, and speed in relation to the virtual fence-designated polygon boundary. As an animal approaches this boundary, the collar triggers a gradually intensifying audio pitch that indicates proximity to the virtual boundary (https://www.nofence.no/en/, accessed on 20 February 2022). If the animal stops moving or changes direction, the audio signal ceases, and no electric pulse is administered. Conversely, if the animal persists toward the virtual fence boundary, the audio tone is succeeded by a mild electric pulse to deter further encroachment [3,4,5].

One of the key advantages of virtual fencing technology is its flexibility in defining land and forage resource allocations using customizable polygons in terms of shape, size, and duration. This flexibility empowers land managers with unprecedented control over grazing practices, addressing the challenge of managing heterogeneous resources across both space and time [6,7,8]. The potential applications of virtual fencing are expansive, encompassing immediate benefits such as the prevention of unwanted access in ecologically sensitive areas, including riparian zones, while concurrently reducing labor requirements [1,4].

Although concerns about animal welfare have arisen regarding virtual fence applications, many of these worries can be attributed to a lack of understanding of the technology’s operational principles. Notably, the core similarity between electric fencing and virtual fencing lies in their use of visual (electric fence) or acoustic (virtual fence) cues to signal the deterrent electric pulse [9,10]. In addition, animals readily adapt to these cues, mitigating potential negative effects on their comfort, well-being, and overall welfare [9,11,12].

Successful implementation of virtual fencing technology hinges on a suitable training phase [4,13,14,15] for the animals to anticipate and move away from the virtual fence boundaries to avoid the electric cue that acts as a physical deterrent. This learning process involves two conditional behavioral mechanisms. The first mechanism, known as the skin defense system, enables animals to steer clear of landscape locations associated with prior exposure to an electric pulse [12,16]. The second mechanism involves associative learning, where animals associate an audio tone with signaling an impending mild electric pulse [8,17,18]. Consistent timing of the device’s delivery of an electric pulse following the audio tone is critical to providing the animal with the necessary predictability to alter foraging trajectories. The gradually intensifying audio pitch as the collar progresses towards the virtual fence boundary serves as a cue for animals to anticipate the onset of the electric pulse. This associative learning also enables animals to adapt to periodic changes in virtual fence boundaries.

Despite prior research efforts, the scientific literature highlights significant knowledge gaps in the development of efficient, effective, and safe animal training protocols for virtual fence applications. Moreover, establishing standard training protocols can serve as benchmarks for future research on virtual fencing, facilitating cross-study comparisons and enabling validation of the application across varying equipment and animal physiological and behavioral differences.

The primary aim of this study was to investigate the virtual fence application, with an emphasis on the learning process associated with the two conditional behavioral mechanisms in a group of nursing Brangus cows (*n* = 28). These cows were equipped with commercial Nofence virtual fence collars (https://www.nofence.no/en/, accessed on 20 February 2022) and utilized an existing ranch infrastructure. In addition, the research outlines a detailed training protocol for the implementation of a virtual fence system.

Initially, our investigation focused on hypothesis 1, which postulated that animals would acquire the ability to avoid restricted zones located outside the virtual fence-designated polygon area by increasing their utilization of the containment zones within the virtual fence-designated polygon area. To explore this hypothesis, we analyzed the GPS coordinates collected using the collars to assess how the animals utilized the spatial dimensions of the arena. Furthermore, we utilized behavioral metrics derived from the collar data to validate the animal’s responses (daily travel distances, exploration of areas, and activity levels) to changes in the allocated area with the virtual fence application.

Subsequently, hypothesis 2 was examined to determine if animals received progressively fewer audio-electric cues over time (Period) and increasingly relied on auditory cues to modify their behavior, in accordance with the associative learning mechanism. This facet of the study involved the evaluation of the number of audio and electric cues received by the animals. Two computations were used to evaluate the relationship between these cues: (1) the ratio of electric to audio cueing and (2) the percentage of electric cues relative to the total number of cues emitted by the collar. Also, the duration of audio cues emitted by the collars was examined to gauge the total length of the audio tone played and to track variations in the length of the emitted audio tone per cue.

## 2. Materials and Methods

### 2.1. Animal and Virtual Fence Collars

All procedures were approved by the New Mexico State University Institutional Animal Care and Use Committee (Protocol 2021–010). Cows with calves over 1.5 months of age were used in the training phase to mitigate challenges associated with younger calves, as recommended by Nyamuryekung’e et al. [19]. Two groups of cows were used in the training phase (*n* = 11 and 17, respectively). These groups were organized based on the calving dates (ranging from 21/2 to 11 April 2022 for Group 1 and from 14/3 to 20 May 2022 for Group 2) to ensure the average initial calf age was approximately 2 months (1.96 vs. 2.25 months for Groups 1 and 2, respectively) at the beginning of the deployment (11 May 2022 and 6 July 2022 for Groups 1 and 2, respectively).

Within their respective groups, nursing Brangus cows that were naïve to the virtual fence technology were selected. These cows had approximate mean weights of 490 kg in Group 1 and 402 kg in Group 2. Cows were equipped with C2 Nofence virtual fence collars (Batnfjordsøra, Norway), each weighing 1.45 kg (https://www.nofence.no/en/, accessed on 20 February 2022) two weeks before the virtual fence training. Collars operated on solar and battery energy, using LTE Cat-M1 or 2G network (cellular) to register virtual fence polygons and communicate real-time animal positions and activity at 15 and 30 min intervals, respectively, with reliable cellular coverage and in near real-time when cellular coverage was suboptimal. During this two-week period of acclimatization, the cows wore the collars while grazing freely alongside their uncollared offspring in pastures near the New Mexico State University’s Chihuahuan Desert Rangeland Research Center headquarters (32°31′53.2′′ N 106°48′15.6′′ W). It is important to note that no virtual fence cueing was initiated during this two-week adaptation Period, and electric fence lines were not employed.

The virtual fence polygons generated using the Nofence device delineated three distinct spatial areas:

- Containment Zones: these areas resided within the virtual fence polygon, granting collared animals the freedom to move without restrictions;
- Cueing Zone: Positioned approximately 5–10 m wide from the containment zone’s edge or virtual fence boundary, this area progressively subjected collared animals to three pairings of audio-electric cueing. Following each cue, an alert was sent to the manager;
- Restricted Zone: Also referred to as the escape zone, these areas were located outside the virtual fence polygon. While collars continued to record data in this zone, no cueing was initiated. In cases of breaches, escape notifications were dispatched to the manager.

Within the cueing zone, the pitch of the audio signal increased progressively, culminating in the highest pitch signal at 82 dB. The complete audio ramp cycle lasted between 5 and 20 s, signaling the administration of the electric pulse upon cycle completion. The actual duration of the audio cueing cycle varied depending on the speed of the animal’s movement as it approached the virtual fence boundary.

Virtual fence boundaries, unlike fixed physical fence lines, exhibit variability due to inherent GPS position errors. The Nofence collars exclusively accept GPS positions with an accuracy of 3.5 m or better for cue initiation. The collars were deactivated safely in case of failure or after emitting three electric pulses in close proximity. Additionally, the Nofence collars featured directional cueing, meaning that animals received cues only when leaving the virtual fence containment zone. Hence, animals were permitted to re-enter the virtual fence polygon without encountering any cues. However, once inside, the collars were reactivated to deter any further attempts to exit the containment zone.

### 2.2. Training Deployment Design

**Figure 1:**
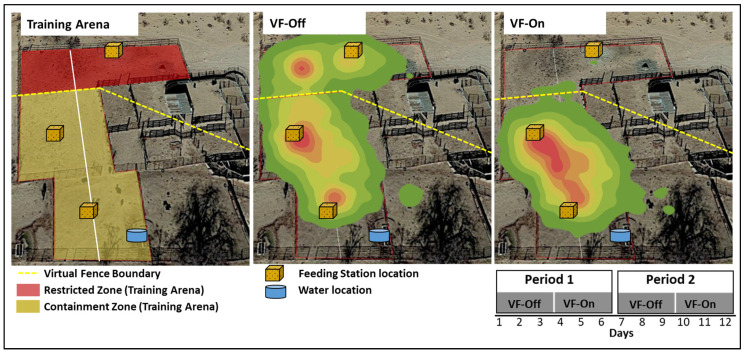
Top left: Training arena (0.2 ha) devoid of vegetation, with three permanent feeding stations spaced along the major axis (white line, 65.80 m), with a single water trough location. The yellow dashed line indicates the virtual fence boundary encapsulating the southern portion of the training arena, delineating it into a containment zone (amber area, southern portion of the training arena, 0.12 ha) and a restricted zone (vermilion area, northern portion of the training arena, 0.08 ha). Heat maps, generated using the kernel density tool with 7 quantile classification categories, depict the spatial distribution (ranging from green as low to red as high) derived from GPS locations of Brangus cows from both groups (*n* = 11 and 17) within the training arena wearing virtual fence-deactivated (middle: VF-Off) or virtual fence-activated (right: VF-On) collars. Bottom right: training deployment design across the two Periods.

At the onset of the training phase, the first group of virtual fence-collared cows (*n* = 11) with uncollared calves (11 May 2022) were moved to a holding pen situated at the New Mexico State University’s Chihuahuan Desert Rangeland Research Center headquarters. The pen (0.2 hectares and devoid of vegetation) contained three permanent feeding stations spaced evenly within each third of the area. Baled, beardless wheat hay (approximately 590 kg) was provided *ad libitum*. Additionally, a single water trough was placed on one side of the pen (Figure 1). Throughout the deployment, to facilitate replenishing of the feeding stations when near depletion, the animals were temporarily moved to the southwestern pen area, and access to the arena was restricted for approximately one hour.

Collared cows were initially allowed to feed freely at the three feeding stations for three consecutive days while wearing virtual fence-deactivated collars (VF-Off). On the fourth day, all collars were configured with a virtual fence polygon drawn to restrict the collared cows from accessing the farthest 40% of the pen area, designating it as the restricted zone (0.08 hectares), while allowing access to the two remaining feeding stations and the water trough location, defined as the containment zone (0.12 hectares). This virtual fence configuration was maintained for three consecutive days (VF-On). The activation or deactivation of the virtual fence configuration occurred at approximately 07:00 am during the initiation of the 3-day interval when all animals were situated within the containment zones. This sequence of three days with virtual fence-deactivated collars (VF-Off), followed by three days with virtual fence-activated collar (VF-On) configuration, was repeated for a second 6-day Period (total duration of the training phase = 12 days) (Figure 1). The same pens and training protocol described above were replicated with a second group of naïve collared cows (*n* = 17) with uncollared calves (6/7/2022).

### 2.3. Data Processing

At the end of the deployment (24 May 2022 for Group 1 and 19 July 2022 for Group 2), data from the Nofence collars were retrieved as CSV files using an online portal from the Nofence website service. The data included time stamps for variables processed by each collar registering the animal positions (latitude, longitude, horizontal accuracy, horizontal dilution of precision, and number of satellites), accelerometer-based activity count, ambient environmental condition (humidity, pressure, and temperature), communication variables (global system for mobile communication operator and communication strength), and collar status (battery voltage, solar charge, VF status, and polygon ID). In addition, the collars registered a time stamp and position for each cueing event, either audio (including duration of the signal) or electric pulse, and escape notifications.

Data were screened for duplicates and filtered to include only data from the training phase for both groups, which occurred from 11 May 2022 to 24 May 2022 for Group 1 and from 6 July 2022 to 19 July 2022 for Group 2. Furthermore, data collected an hour before and after the virtual fence configuration status changed (06:00 am–08:00 am) and from when the animals were temporarily relocated to an adjacent pen area (~one hour) for feeding station refill were excluded from the analysis.

To facilitate the analysis, a virtual fence status column (VF-On or VF-Off) was created, categorizing the data into segments representing the three consecutive days. The virtual fence polygon used during the VF-On status and the position data collected from the collars were imported and projected to the NAD 1983 UTM Zone 13N coordinate system using ArcGIS software (ESRI 2018, ArcMap Desktop v. 10.6). Position data from each collar were overlaid onto the virtual fence polygon layer to extract precise location information pertaining to either being on the containment or restricted zones. Data from each collar were processed by day to include the percentage of time (%Time) GPS location data were within the containment and restricted zones during both virtual fence deactivated (VF-Off) and virtual fence activated (VF-On) status.

Furthermore, from projected GPS coordinate data, the daily distance traveled per collar (Dist) was calculated using the Pythagoras theorem applied to sequential GPS coordinates within a day. To assess the daily area explored by each collared animal (Area), the Minimum Bounding Geometry tool (ESRI 2018, ArcMap Desktop v. 10.6) was employed, which generated a polygon encompassing the minimum area containing all GPS coordinates recorded within a given day [19,20]. In addition, accelerometer-based activity data from the Nofence CSV files, represented as a count of motion intensity using internal threshold values within 30 min intervals, were aggregated into a cumulative count within a day (#Activity).

Variables related to collar cueing events were processed to include the daily count of audio (#Audio) and electric pulses (#Electric). Additionally, two derived variables were computed: one representing the ratio of electric to audio cueing per day (Electric/Audio), and the other indicating the percentage of electric cues relative to the total number of cues emitted by the collar per day (%Electric/Cue; equates to (#Electric/(#Electric + #Audio)) × 100). As for the duration of the audio signal, two daily variables were generated, including the sum of the total daily audio signal length (Sum_DurAudio) and the average length of a single audio cue (Avg_DurAudio). Notably, both variables related to the duration of the audio signal were computed exclusively when a registered audio warning was present.

### 2.4. Data Analysis

To prepare the data for statistical analysis, all daily variables related to spatial analysis (%Time, Dist, and Area) and activity (#Activity) of the animals, as well as variables related to cueing events (#Audio, #Electric, Electric/Audio, %Electric/Cue, Sum_DurAudio, and Avg_DurAudio), were averaged for each cow over three-day Periods, aligning with the concurrent virtual fence status (VF-On or VF-Off). This approach not only helped smooth the dataset by mitigating the daily variability inherent in biosensing data but also addressed the issue of an unequal number of sampling days [20]. This disparity was particularly evident in the case of cueing event data, for which there were instances of VF-On status within a day when several animals did not interact with the virtual fence boundary. For instance, when analyzing data on a daily scale, the percentage of days with no cueing events was 66.2% (59.1% in Period 1 and 73.6% in Period 2). However, with Period averaging, the percentage of data points with no cueing events decreased to 25.5%, with 21.4% in Period 1 and 29.6% in Period 2.

The averaged daily variables related to spatial analysis (%Time, Dist, and Area) and activity (#Activity) were subjected to statistical analysis using SAS 9.3 software (SAS Institute, Cary, NC, USA). The PROC MIXED procedure, along with a “covtest” statement, was employed for the analysis. Analysis of Variance with the Kenward–Roger degrees of freedom statement was used to model the effects of virtual fence status (VF-On vs. VF-Off), the Period (1 vs. 2), and their interaction on the averaged daily variables related to spatial analysis (%Time, Dist, and Area) and activity (#Activity) of the animals. Random effects were considered for Groups (1 and 2) and CollarID (*n* = 11 and *n* = 17 for Groups 1 and 2, respectively) to account for variations associated with these factors. Least squares means were calculated for the main effects of the virtual fence status and Period individually, with the “pdiff” statement used for pairwise comparisons. Furthermore, using the least squares means computation for the interaction effect, the virtual fence status effect (VF-On vs. VF-Off) within the Period was examined using the “slice” statement.

Descriptive analysis was initially conducted on the raw cueing event data, which included #Audio and #Electric from the collar dataset. Similarly, the averaged daily variables related to cueing events (#Audio, #Electric, Electric/Audio, %Electric/Cue, Sum_DurAudio, and Avg_DurAudio) were analyzed using SAS 9.3 software (SAS Institute, Cary, NC, USA). However, this analysis focused exclusively on the dataset that considered virtual fence-activated status (VF-On). Employing the PROC MIXED procedure with a “covtest” statement, Analysis of Variance was carried out with the Kenward–Roger degrees of freedom statement, focusing on the effects of the Period (1 vs. 2) on the averaged daily variables associated with cueing events. Random effects were incorporated for Groups (1 and 2) and CollarID (*n* = 11 and *n* = 17 for Groups 1 and 2, respectively). The main effect of the Period was assessed via computing least squares means, and pairwise comparisons were conducted using the “pdiff” statement. In all analyses, statistical significance was declared at *p* < 0.05.

## 3. Results

**Figure 2:**
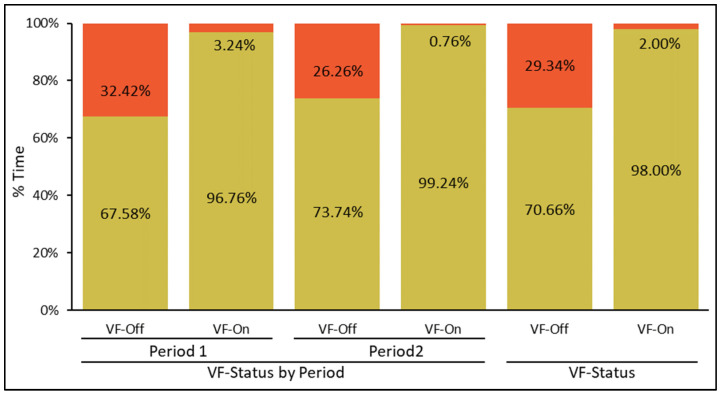
Analysis of the percentage of time spent by virtual-fence-collared nursing Brangus cows within the containment (amber bars) and restricted (vermilion bars) zones across the two different Periods under varying VF-Status (virtual fence deactivated (VF-Off) vs. virtual fence activated (VF-On)). The figure also includes an overall comparison of VF-Status (VF-Off vs. VF-On) as the main effect (represented by the two bars on the right, VF-Status). Significance testing indicates that all pairwise comparisons of VF-Status (VF-Off vs. VF-On) were statistically significant for the percentage of time allocation within either the containment or restricted zones.

The percentage of time collared animals spent within the containment and restricted zones was significantly influenced by their main effects, namely the virtual fence status (*p* < 0.01) and Period (*p* < 0.01), with no significant interaction effect (*p* = 0.10). When considering the main effect of virtual fence status, VF-On increased the percentage of time collared animals spent within the containment zones (98.0 vs. 70.7 ± 1.2% Time; *p* < 0.01) while reducing the percentage of time within the restricted zones (12.0 vs. 29.3 ± 1.2% Time; *p* < 0.01) compared to VF-Off status (Figure 2). In terms of the main effect of the Period, which encompassed 6 concurrent days (3 days of VF-Off followed by 3 days of VF-On), Period 2 resulted in a higher percentage of time collared animals spent within the containment zones (86.5 vs. 82.2 ± 1.2% Time; *p* < 0.01) and a lower percentage of time within the restricted zones (13.5 vs. 17.8 ± 1.2% Time; *p* < 0.01) compared to Period 1 (Figure 2).

Similar trends were observed for the remaining variables related to spatial analysis (Dist, Area) and activity (#Activity) of the animals, where significant main effects were detected for the virtual fence status (Dist; *p* < 0.01, Area; *p* < 0.01, and #Activity; *p* < 0.01) and Period (Dist; *p* < 0.01 and #Activity; *p* < 0.01), except for Area (*p* = 0.31). The interaction effect was not significant for these variables (Dist; *p* = 0.15, Area; *p* = 0.06, and #Activity; *p* = 0.19) (Table 1). For the main effect of virtual fence status, VF-On resulted in a decrease in the daily distance traveled (648.0 vs. 883.8 ± 82.7 m; *p* < 0.01), area explored within the arena (1193.8 vs. 1982.1 ± 121.4 m^2^; *p* < 0.01), and the cumulative activity count of the animal (14,988 vs. 17,300 ± 1335.5; *p* < 0.01) compared to the VF-Off status. Regarding the main effects of the Period, Period 2 exhibited lower values for the daily distance traveled (719.0 vs. 812.7 ± 82.7 m; *p* < 0.01) and the cumulative activity count of the animal (15,330 vs. 16,958 ± 1335.5; *p* < 0.01) when compared to Period 1.

**Figure 3:**
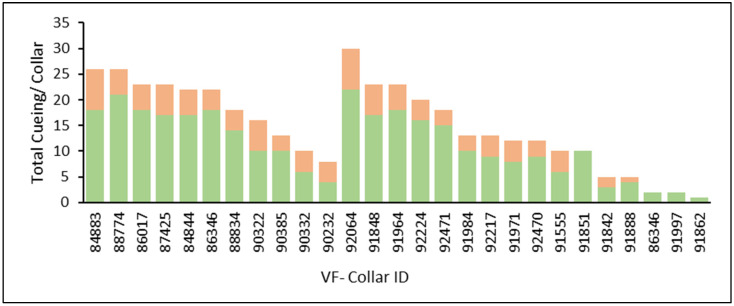
Ranking of total auditory (green) and electrical pulses (orange) emitted per cow during the training phase for naïve nursing Brangus cows from two groups (*n* = 11 and 17).

The cueing events during the training deployment included 305 audio tones (153 for Group 1 and 152 for Group 2) and 101 electric pulses (54 for Group 1 and 47 for Group 2) in the two groups of nursing Brangus cows (Figure 3). The total number of cueing events per collared cow within the two groups ranged from 8 to 26 for Group 1 and 1 to 30 for Group 2 (Figure 3).

The daily number of emitted audio and electric pulses was significantly influenced by the Period (#Audio; *p* < 0.01 and #Electric; *p* < 0.01). During Period 1, when the virtual fence was first activated, individual cows experienced a higher frequency of auditory (1.9 vs. 0.6 ± 0.4; *p* < 0.01) and electrical cues (0.7 vs. 0.2 ± 0.2; *p* < 0.01) compared to Period 2. In terms of variables representing the animals’ reliance on audio warnings, the ratio of electric to audio cues per cow (Electric/Audio) was not significant for the Period (*p* = 0.10). However, the percentage of electric cues relative to the total number of cues emitted by the collar differed between Periods (%Electric/Cue; *p* = 0.04). During Period 1, exposure to the virtual fence activated status resulted in a higher percentage of electric cues relative to the total cues per cow (23.0 vs. 13.3 ± 3.8%; *p* = 0.04) compared to Period 2. Lastly, regarding the duration of the audio signal, the length of the sum of the total daily audio signals (Sum_DurAudio) differed between Periods (*p* = 0.02), while the average length of a single audio cue (Avg_DurAudio) did not differ between Periods (*p* = 0.91). Animals in Period 1 were exposed to longer durations within the day with collars emitting the audio warning (58.2 vs. 26.7 ± 8.7 sec; *p* = 0.02) compared to Period 2 (Table 2).

## 4. Discussion

In this study, we set out to investigate hypothesis 1, that animals would develop the ability to avoid restricted zones outside the virtual fence-designated polygon area by increasing their use of the containment zones (skin defense mechanism). Our findings support this hypothesis. Virtual fence activation significantly reduced the percentage of time animals spent within the restricted zones while increasing the time spent within the containment zones. In essence, during the training phase, virtual fence activation successfully contained the cows within their designated zones approximately 98.0% of the time, consistent with other findings [9,10,11,12,13,21]. Additionally, the significant main effect of the Period further suggests an ongoing learning process among the cows regarding the virtual fence polygon’s configuration. Notably, with regard to the escape notifications derived from collar data, which indicated instances when animals breached the containment zone, we observed 10 such notifications, all occurring in Period 1, involving three animals in Group 1 and a single animal in Group 2, while Period 2 had no recorded escape events.

Furthermore, our study leveraged behavioral metrics derived from collar data, specifically focusing on daily travel distance, area exploration, and accelerometer-based activity levels. These metrics provided valuable insights into how the animals responded to changes in the allocated area imposed by the virtual fence application. Activation of the virtual fence led to a reduction in all these behavioral metrics, as the animals were confined to 60% of their initial designated pen area (0.12 hectares out of 0.2 hectares). However, the reduction in the activity variable during virtual fence-activated status may also suggest some level of adjustments in the animal’s time budget, as observed in other studies [9,21,22]. This aspect of the result warrants further investigation.

In addition, the behavior metrics displayed Period-dependent effects, with the second Period showing lower values compared to the initial Period. This adjustment can be attributed to the following rationale: as the second Period began with VF-Off status for three days, the animals displayed a delayed response from the previous three days of VF-On status in the first Period. This delayed response of the animals when exploring previously restricted zones might be associated with animals taking time to become familiar with the arena area or waiting for their peers to venture into the restricted zones before following suit [23]. However, during the VF-On status of the second Period, the animals drew upon their prior learning experiences from the first period to correctly adjust their behavior, resulting in lower behavior metrics compared to the VF-On status of the first Period.

The real-time data collected from sensors integrated into virtual fencing devices hold great promise for economical and effective animal monitoring [1,2,24,25]. These data can be tailored to address questions related to spatial use (animal position) [20,26], activity patterns (motion sensors) [27,28], and even ambient environmental conditions (humidity, pressure, and temperature). This enables a detailed and accurate analysis of the animals’ operational status. The integration of such information, harnessed from virtual fence sensor data, provides invaluable support for the surveillance and monitoring of animals, enhancing data-driven decision-making processes for land managers in the context of precision livestock farming within rangeland management [24,25]. The sensitivity of the behavioral metrics derived from our dataset further underscores the potential of virtual fence collar data for animal surveillance and monitoring within precision livestock farming applications.

Our study provides empirical support for Hypothesis 2, which proposed that animals would gradually receive fewer audio-electric cues over time and would increasingly depend on auditory cues to modify their behavior through associative learning. We indeed observed a clear reduction in the frequency of audio-electric cues over time, corroborating this hypothesis. Specifically, during Period 2, when the virtual fence was activated, cows received notably fewer auditory and electrical cues compared to Period 1. This observed reduction is consistent with findings reported in previous studies on virtual fence applications [10,11,12,21,29]. The reduction during Period 2, as quantified using the equation ((1 − (Period 2/Period 1)) × 100), was 66% for auditory and 76% for electric cues. Additionally, the observed numerical difference in the reduction rates during Period 2 lends support to the notion that animals increasingly rely on auditory over electric cues as they become more familiar with the virtual fence system.

In parallel with the cueing results, the variables assessing animals’ reliance on audio warnings, specifically the ratio of electric to audio cueing per cow, were not significant for Period. This is in contrast to Lomax et al. [11], who reported a decrease in the ratio of electric to audio cueing. However, a significant reduction in the percentage of electric cues relative to the total number of cues was evident, with animals receiving a smaller proportion of electric cues relative to the total in the second Period. The inconsistency between the results of these two variables could be attributed to their differing sensitivity levels as well as the averaging of the variables across Periods before statistical analysis. Lomax et al. [11] reported differences in the ratio of electric to audio cueing on a daily scale, with statistical differences only present on the first day of animals encountering the virtual fence configuration.

Lastly, regarding the duration of the audio signal, animals in Period 1 experienced longer durations during the day when collars emitted audio warnings compared to Period 2. This finding corroborates the total number of audio warnings emitted across the two Periods. However, the lack of significance in the average length of a single audio warning was unexpected. The collars were programmed to deliver audio warnings progressively with varying pitches, ultimately reaching the highest pitch signal at 82 dB, designed to help animals learn when the electric pulse would be emitted. However, our results suggest that this progressive pitch strategy might not have had the expected impact. This finding suggests the need for further investigation in future research to decipher the most suitable audio signal or whether animals respond consistently as long as there is an audio cue.

The individual variation among animals in total cueing events, as revealed in the descriptive analysis, presents an intriguing opportunity for further investigation of virtual fence applications through individual herd selection to address animal welfare concerns. However, breed and within-breed individual differences related to both social- and asocial-learning mechanisms could potentially influence an individual’s ability to adapt to the technology [11,13,14,21,29].

The concept of selectively using virtual fence collars on specific individuals has sparked interest in its potential applications for large herds. However, empirical support for this idea has been limited, with only a single study conducted on sheep suggesting that virtual fence collaring the entire herd, or 66% of it, would yield comparable results [22]. In our study, we employed mother–offspring pairings during the training phase, with only the cows wearing the virtual fence collars. While the recommended approach for effective virtual fencing is to collar all individuals within a herd continuously, regardless of their social rank, as advised by Keshavarzi et al. [13], our research demonstrates the effective containment and learning capabilities of the dams, regardless of the behavior of their offspring. Informal observations revealed that, on occasion, some calves ventured into the restricted zones during virtual fence-activated status. However, this behavior was not systematically monitored, presenting an opportunity for further research investigations.

Cattle are gregarious in nature; hence, learning about novel situations can be facilitated through their conspecifics [13]. In the group deployment, this response was noted through the anecdotical observation of the reaction of a neighbor cow to the cues emitted from a peer’s collar and was also corroborated by the other literature [29]. In addition, the gregarious nature of cows may encourage cued individuals to respond appropriately during training by re-joining herd members inside the containment zone when challenged with the tactile stimulus, imitating a predatory response (skin defense mechanism). As a precaution to the cued individuals’ reactions during the training phases, adjacent non-collared cows outside the training arena at the headquarters pen location were restricted to areas behind the containment zone. This training approach extends to pasture deployment, as cautioned by Verdon et al. [23], who recommended avoiding the use of virtual fence applications for managing cattle with close visual contact with other herds.

However, to facilitate proper animal training for the virtual fence application, it is advised that all collared individuals receive at least a single cueing event [14]. Our training deployment design could facilitate this by incorporating additional Periods within the deployment with the alternative virtual fence-deactivated (VF-Off) status configuration followed by the virtual fence-activated (VF-On) status configuration. Moreover, our choice of a 3-day sequence for the virtual fence configuration demonstrated effectiveness in our training deployment. However, factors such as animal density (*n* = 11 and *n* = 17 for Groups 1 and 2, respectively) within the containment zone (0.12 ha) and the ratio of the virtual fence boundary (23.87 m) to the physical fence boundary (139.45 m) within the containment zone perimeter are noteworthy variables that should be thoroughly investigated to determine the optimal duration for the sequencing of virtual fence configurations.

The deactivation status of the virtual fence plays a crucial role in establishing a baseline for the virtual fence application. The initial period, commencing with virtual fence deactivated status (VF-Off), serves as a control to assess the effectiveness of the virtual fence application. In our training phase setup, with three feed stations providing baled hay evenly distributed across each third of the arena area, the animals spent approximately 67.6% of their time in the containment zone, which was roughly 60% (0.12 ha) of the area, and 32.4% of their time in the restricted zone, equivalent to the remaining 40% (0.08 ha) of the arena area. The assessment of the selectivity of the containment versus restricted zone using Ivlev’s electivity index (E = ((r − p)/(r + p)), where r is the proportion of time allocated within a zone and p is the proportion of the zone area within the arena, yielded a value of 0.06 for the containment zone and −0.10 for the restricted zone [20,30]. This indicates that, at the start of the training phase, due to the elective index being closer to 0, animals had indifferent selectivity for the containment and restricted zones, substantiating the effectiveness of the virtual fence application. Furthermore, establishing this baseline can provide insights into variations in cueing rates across different virtual fence studies by defining the preference for the restricted area before application, hypothesizing that a higher preference may result in more cueing. Additionally, the second period, commencing with VF-Off status, serves as a control to ensure that animals do not associate spatial location with cueing but rather the presence of the audio cue as a warning of an impending mild electric pulse. The inclusion of a single water trough location within the containment zone further enhanced training success, as animals that ventured beyond the containment zones could be recaptured with the system when prompted by thirst. Consequently, the training protocol presented here offers an efficient, effective, and safe approach to animal training for virtual fence applications.

## 5. Conclusions

Our results indicate that groups of cows learn rapidly to respond to VF boundaries by reducing time spent within the restricted areas and minimizing the frequency of cueing events to alter behavior. Assuming animals complete the training phase successfully, adverse effects are minimized as animals learn to respond to the audio and avoid the mild electric cue, which minimizes adverse effects on animal comfort, well-being, and welfare. In addition, the associative learning process enables animals to adjust readily and respond to periodic changes in virtual fence boundaries, providing managers with the flexibility to allocate land and forage resources with polygons of configurable shape, size, and duration.

## Figures and Tables

**Table 1:** Least square means estimates and their standard errors (SE) for the interaction term (virtual fence status (VF-Status) × Period) illustrating daily travel distances, area exploration, and activity levels in virtual-fence-collared nursing Brangus cows. The *p*-values indicate the significance of the main effects of VF-Status (virtual fence deactivated collar status (VF-Off) vs. virtual fence activated collar status (VF-On)), Period (representing two 6-day Periods with each composed of 3 days of VF-Off followed by 3 days of VF-On), and the interaction effect.

	Period 1		Period 2		SE	*p*-Value		
	VF-Off	VF-On	VF-Off	VF-On		VF-Status	Period	Interaction
Dist (m)	946.28	679.19	821.31	616.75	84.06	<0.01	<0.01	0.14
Area (m^2^)	1956.87	1277.30	2007.40	1110.25	128.01	<0.01	0.31	0.06
Activity	18,406	15,510	16,194	14,465	1371.71	<0.01	<0.01	0.19

**Table 2:** Least square means estimates with standard errors (SE) for Period, illustrating the daily count of audio and electric pulses, the ratio of electric to audio cueing (Electric/Audio), the percentage of electric cues relative to the total number of cues (%Electric/Cue), the sum of the total daily audio signal length (Sum_DurAudio), and the average length of a single audio cue (Avg_DurAudio) emitted per virtual fence-collared nursing Brangus cows. The *p*-values denote the significance across Periods, representing 3 days of virtual fence-activated status.

	Period		SE	*p*-Value
	1	2		
#Audio	1.88	0.64	0.45	<0.01
#Electric	0.70	0.17	0.17	<0.01
Electric/Audio	0.36	0.22	0.08	0.10
%Electric/Cue	23.02	13.31	3.76	0.04
Sum_DurAudio (sec.)	58.16	26.70	8.74	0.02
Avg_DurAudio (sec/audio.)	11.87	11.66	1.38	0.91

## Data Availability

The raw data concerning the virtual fence sensors showcased in this study are available upon request from the corresponding authors.
